# Quantitative method for resilience assessment framework of airport network during COVID-19

**DOI:** 10.1371/journal.pone.0260940

**Published:** 2021-12-03

**Authors:** Jiuxia Guo, Yang Li, Zongxin Yang, Xinping Zhu

**Affiliations:** 1 School of Air Traffic Management, Civil Aviation Flight University of China, Guanghan, Sichuan, China; 2 Operation Supervisory Center, Civil Aviation Administration of China, Beijing, China; University of Shanghai for Science and Technology, CHINA

## Abstract

The resilience and vulnerability of airport networks are significant challenges during the COVID-19 global pandemic. Previous studies considered node failure of networks under natural disasters and extreme weather. Herein, we propose a complex network methodology combined with data-driven to assess the resilience of airport networks toward global-scale disturbance using the Chinese airport network (CAN) and the European airport network (EAN) as a case study. The assessment framework includes vulnerability and resilience analyses from the network- and node-level perspectives. Subsequently, we apply the framework to analyze the airport networks in China and Europe. Specifically, real air traffic data for 232 airports in China and 82 airports in Europe are selected to form the CAN and EAN, respectively. The complex network analysis reveals that the CAN and the EAN are scale-free small-world networks, that are resilient to random attacks. However, the connectivity and vulnerability of the CAN are inferior to those of the EAN. In addition, we select the passenger throughput from the top-50 airports in China and Europe to perform a comparative analysis. By comparing the resilience evaluation of individual airports, we discovered that the factors of resilience assessment of an airport network for global disturbance considers the network metrics and the effect of government policy in actual operations. Additionally, this study also proves that a country’s emergency response-ability towards the COVID-19 has a significantly affectes the recovery of its airport network.

## 1. Introduction

Large-scale disruptive events, which include unfavorable weather, failures of certain network components, industrial actions of air transport staff, natural disasters, terrorist threats/attacks, and traffic incidents/accidents, can jeopardize the resilience and vulnerability of an air transport network [1]. An air transport network comprising airports and airline flights scheduled can be affected by the abovementioned disruptive events. Resulting in extended airline flight delays and cancellations. To reduce social and economic losses caused by disruptive events, the resilience and vulnerability of the air transport network must be investigated, such that the network can recover rapidly and efficiently recover to normality after a disruptive event. Resilience refers to a system’s intrinsic ability to adjust its functioning before, during, or after changes and disturbances [2]. The current COVID-19 pandemic demonstrated that disruptive events can affect transportation networks, particularly air traffic, in terms of the safety and security of airlines, airports, and air traffic control departments. As compared with business pre-COVID-19, a loss of 64.6% (6121 million people) in terms the number of international and domestic passengers and 65% in airport revenues (approximately USD 125 billion) have been record in 2020 based on data from Airports Council International (ACI) data [3].

Numerous studies pertaining to resilience have been conducted for different fields. Holling proposed the resilience concept in ecological systems [4]. Subsequently, the concept of resilience developed in disciplines such as ecology, psychology, organization, social, and engineering was classified into four categories: resilience as rebound, resilience as robustness, resilience as gracefully extensibility, and resilience as sustained adaptability [5]. Compared with other terms, such as robustness, reliability, survivability, and flexibility, resilience focuses on performance degradation and recovery after inevitable disruptions [6]. In recent decades, most studies pertaining to qualitative resilience assessment approaches included conceptual frameworks and semiquantitative indices [7]. Resilience has been extensively investigated in transportation systems, such as railway networks, road networks, and air transport networks [8–10], where natural hazards or extreme weather are typically involved [11, 12]. Research pertaining to the resilience of the air transport system focuses primarily on the air traffic management (ATM) system and airport network [13, 14]. Cook proposed a cost resilience metric for ATM during disturbance [15]. Faturechi introduced an exact solution methodology based on the integer L-shaped decomposition to assess and maximize the resilience of an airport’s runway and taxiway network under multiple potential damage-meteorological scenarios [16]. Additionally, Wang employed a simulation model that considered structural and dynamical factors to investigate the resilience of airport networks [17]. Zhou developed a resilience metric to measure airport resilience post severe weather [18]. Several researchers have recently adopted a network science approach to analyze the degree of synchronization between the number of cases in certain countries and their reactions to air transportation operations [19, 20]. The authors of [21] investigated the resilience of the Chinese airport network (CAN) affected by global public health events based on historical data and assessed the recovery of Chinese and European airports based on different control strategies.

**Fig 1:**
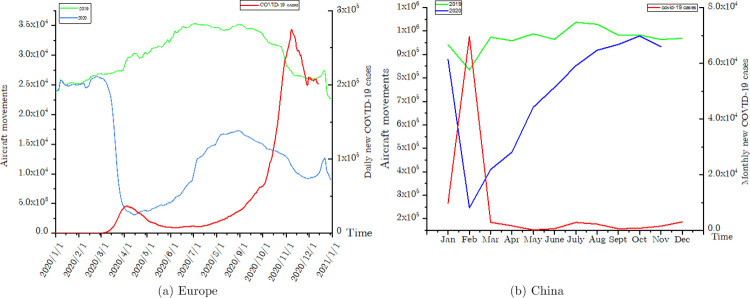
Evolution of air traffic and COVID-19 confirmed cases in Europe and China.

Fig 1 shows that the curve for COVID-19 confirmed cases is inversely proportional to the flight volume curve. In the first half of 2020, the number of aircraft movements at airports in China’s mainland decreased by75% as compared with the previous year. However, after implementing effective prevention and control measures, the number of aircraft movements in June resumed to the level of 2016. Meanwhile, the number of flights in EUROCONTROL member states declined to 54.8% compared with the same period in 2019. Traffic in some European states began declining again following the resurgence of COVID-19 since the beginning of September 2020 (see the blue line in Fig 1A). Confirmed cases of the COVID-19 in Europe continued to increase in the past year (see the red line in Fig 1A). By contrast, the red line in Fig 1B shows that confirmed cases of the COVID-19 in China peaked in February and declined to an extremely low level in May. However, the resilience of airport networks affected by the COVID-19 pandemic is yet to be assessed. Previous studies regarding network resilience primarity focused on the vulnerability and robustness of the network. Therefore, we aim to model the characteristics of airport networks during the pandemic and assess their resilience during the global COVID-19 pandemic globally.

In this study, we focused on the effect of a global-scale disruption on the resilience of airport networks. Nonetheless, the results and insights are also applicable to the effect of combined disruptions (such as epidemics and natural disasters) on airport networks. From a complex network perspective, we analyzed the topological structures of the CAN and European airport network (EAN) and compared vulnerability of the airport network in different countries and regions based on different attack strategies. In addition, from a node perspective, the resilience of individual airports in China and Europe were evaluated, and some improvement strategies to mitigate the disruptions due to the COVID-19 were proposed.

The remainder of this paper is orgazized as follows: Section 2 introduces the method of resilience assessment framework for airport network. Section 3 presents a case study pertaining to the resilience assessment of the CAN and EAN. Finally, Section 4 provides the conclusions and future work.

## 2. Methods

**Fig 2:**
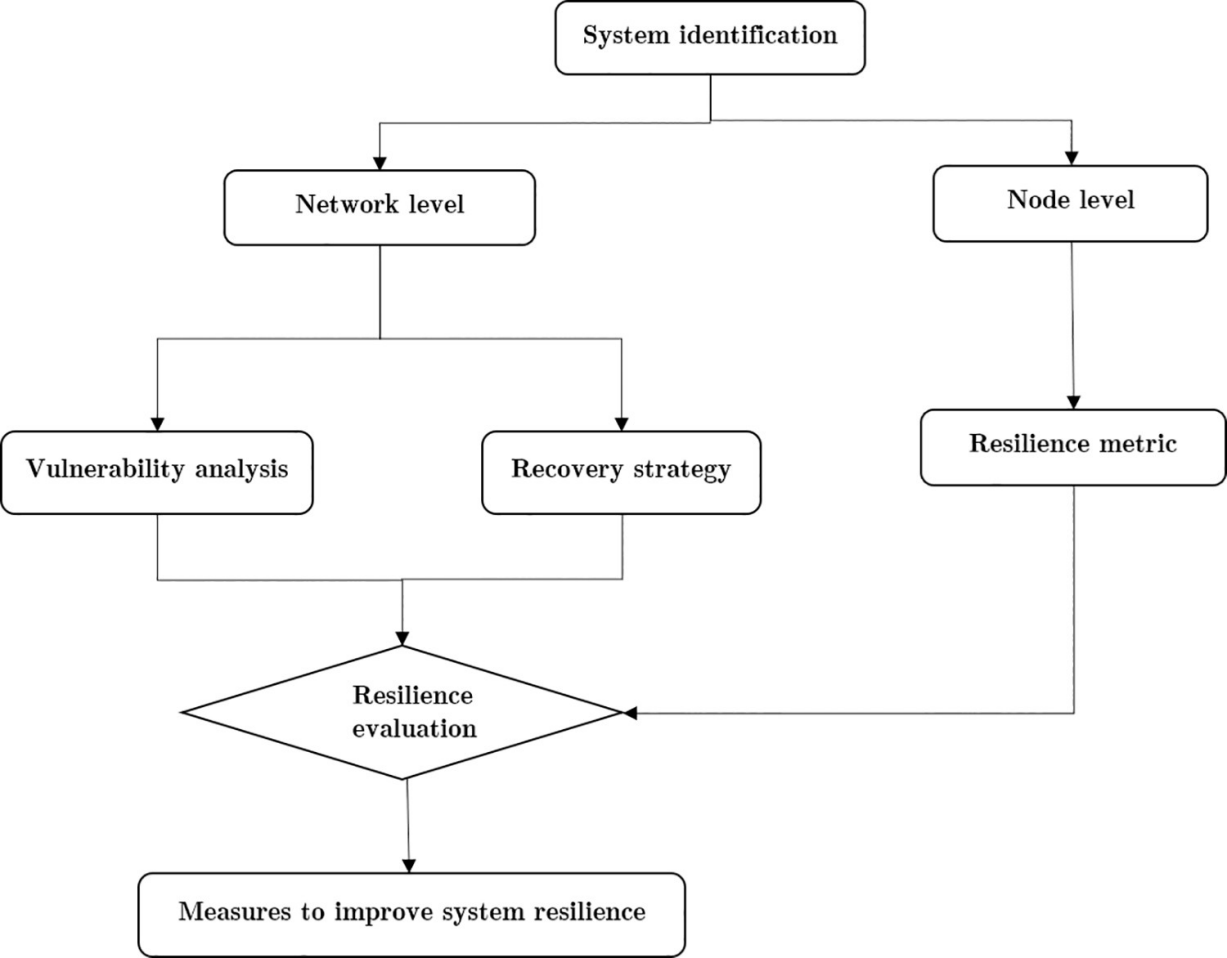
Resilience assessment framework for airport network.

This section introduces a method of the resilience assessment framework for airport networks, including network- and node-level analyses. At the network level, we primarily discuss the vulnerability analysis and the recovery strategy for airport networks. Subsequently, we discuss the resilience metric of individual airports at the node level (see Fig 2).

### 2.1 Airport network modeling

Complex network theory provides effective tools for understanding the structure and dynamics of an airport network system [22]. The airport network is a complex network comprising airports as nodes and the correlations between airport traffic flow as edges; it is widely vestigated at both the international and domestic levels. The air transport networks of some countries such as China, India, Italy, and Australia are small-world networks [23–27]. The metrics for weighting the importance of network nodes include degree, betweenness, and closeness. The heterogeneous structures of airport networks result in different airports possessing different extents of importance. Additionally, the traffic flow distribution is vital to airports. Li [28] combined an airport network’s topological and functional features to evaluate the airport’s importance, based the degree, betweenness, and traffic metrics.

In this study, actual airline data for 232 domestic airports in mainland China and 82 airports in Europe for 2019 were used to form the CAN and the EAN. The key metrics used to characterize the airport network, based on previous studies [22, 25, 28], were used in this study, as shown in Table 1.

**Table 1:** Network metrics.

Parameter	Equation	General Implication
Non-weighted Degree	$k_{i}=\sum_{j=1}^{v}a_{ij}$	*k*_*i*_ refers to the total number of connections node cc has with other nodes in the network. *a*_*ij*_ represents the presence of a link between nodes *i* and *j*,
Weight Degree	$s_{i}=\sum_{j=1}^{N}a_{ij}w_{ij}$	In an airport network, *s*_*i*_ the number of aircraft movements associated with the node represents airport pairs’ edge weight.
Degree distribution	$p(k)=\frac{n_{k}}{n}$	For a network with *n* nodes, *n*_*k*_ is the number of degrees *k*.
Weighted clustering coefficient	$C_{i}^{w}=\frac{1}{G_{i}(k_{i}-1)}\sum_{j,h}\frac{w_{ij}+w_{ih}}{2}a_{jh}$	*G*_*i*_ and *k*_*i*_ is the node weight and node degree of note *i*, respectively.
The average shortest path length	$L=\frac{1}{\frac{1}{2}N*(N-1)}\sum_{i\geq j}d_{ij}$	*d*_*ij*_ is the shortest path length from between nodes *i* and *j*
Betweenness centrality	$B(i)=\sum_{j\neq h}\frac{\sigma_{jh}(i)}{\sigma_{jh}}$	*σ*_*jh*_ is the total number of shortest paths from node *j* to node *h*.
Network efficiency	$E=\frac{1}{N*(N-1)}\sum_{i\neq j}^{N}\frac{1}{d_{ij}}$	The greater the E value, the better the network connectivity. This is global efficiency.

We considered airport networks as directed and weights and regarded the number of aircraft movements between two airports as weights to calculate the critical metrics. The CAN in 2019 included 232 nodes and 2932 undirected edges (the total degree was 5863). The average degree was 25.06, and the network efficiency was 52.08%. The top 8 airports with the highest degrees were Xi’an, Chongqing, Chengdu, Beijing, Kunming, Shenzhen, Shanghai Pudong, and Guangzhou. Compared with the CAN structure presented in [29], the average clustering coefficient decreased, where the number of nodes and average degree increased. In addition, the EAN in 2019 included 82 nodes and 3778 edges (the total degree was 3854). The average degree was 47, the network efficiency was 78.98%, and the average clustering coefficient was 78.38%. Hence, The CAN and EAN are typical small-world networks.

### 2.2 Resilience analysis of airport network

In the transportation system, resilience, vulnerability, and robustness are the most relevant and representative concepts. These concepts are investigated from different perspectives, including network security and system integrity perspectives [9]. The resilience of complex networks is interpreted as the ability to retain performance during and after disruptions and resume to the normal state of operation promptly after disturbances. The properties of social system resilience include robustness, redundancy, resourcefulness, and rapidity. From a system perspective, resilience analysis involves three aspects: the system can resist interferences, absorb interferences, and resume to normality after being disturpted. From a network structure perspective, vulnerability primarily refers to the network’s sensitivity to emergencies [9, 30].

#### 2.2.1 Vulnerability

The perturbation of complex networks include node/link removal, weight reduction, or any of their combination. In this study, the following strategies were adopted to investigate the resilience and invulnerability of an airport network:

*2*.*2*.*1*.*1 Removing nodes randomly*. We randomly selected 5% of nodes in an airport network in each step, removed them, and then analyzed the network’s invulnerability when multiple nodes were attacked simultaneously. Hence, the airport network affected synchronously by different disruptive events (e.g., health emergencies, aviation accidents, and extreme weather) can be simulated.

*2*.*2*.*1*.*2 Removing highest weight nodes*. Nodes were removed based on different weight indicators. We removed nodes in the order of decreasing node degree, betweenness centrality, and node strength [31].

#### 2.2.2 Recovery strategies

From a complex system perspective, the strategies for network recovery after a disruption include using subgraph, degree, path shorten, and random recovery [31]. To simulate the airport network, we applied degree and node strength recovery strategies to manage network attacks, which recovered the nodes in the order of decreasing degree and the node throughput.

From a system operation perspective, the COVID-19 as a global-scale disruptive event; therefore, the recovery strategies and response speed of complex systems are crucial to resume the expected level of the system. Therefore, the strict mobility restrictions and quarantine policies were enforced by countries, which resulted in many flight cancellations and reduced air passenger demand. However, the ongoing vaccination process has resulted in more resilient domestic passenger traffic as compared with international passenger traffic [32]. Based on the current anti-epidemic measures of countries worldwide, we selected two recovery policies to analyze the effect of the COVID-19 on airport networks [33].

*2*.*2*.*2*.*1 Recovery strategy-I*. This strategy involves a unified set of preventive and control policies and strict regulations pertaining to the COVID-19 for supporting airlines and airports recovery. Examples include vaccination, strict quarantine requirements, the lock-down of cities, social distancing, wearing of face mask, mobility constraints, proactive testing, isolation of suspected and confirmed cases, promotion of personal hygiene, and development of an effective contact tracing system.

*2*.*2*.*2*.*2 Recovery strategy-II*. This strategy involves a series of anti-epidemic measures but takes a less strict enforcement policy. Examples include curfews, quarantines, and certain regulations such as stay-at-home orders, shelter-in-place orders, and lock-downs.

#### 2.2.3 Resilience model of airport network

Based on previous studies, resilience is associated with a system withstanding disturbances, adapting to the disruptions, and recovering from the state of deteriorated performance. The analysis of airport network resilience is fundamental for understanding its sustainability, which affects the network operation efficiency [28]. The resilience of the network structures can be quantified in terms of throughput and connectivity or compactness. The deterioration of the nominal performance of airports by the effect of the COVID-19 can reflect their resilience. This paper focuses on the domestic airport network in different countries/regions, where airports and their direct flights. are represented by nodes and links, respectively. The operational resilience of an airport network can be analyzed using resilience metrics [17]. However, the resilience metric of airport network nodes can be assessed based on the traffic volume (node’s strength). The resilience metrics for an airport network’s nodes can be expressed as follows [21].

$$\begin{array}{l} GM_{KPI}=f(R_{KPI},RAPI_{KPI}^{DP},RAPI_{KPI}^{RP},TAPL_{KPI},\;RA_{KPI})\\ =R_{KPI}\times \left(\frac{RAPI_{KPI}^{RP}}{RAPI_{KPI}^{DP}}\right)\times {\left(TAPL_{KPI}\right)}^{-1}\times RA_{KPI} \end{array}$$ (1)

where *R*_*KPI*_ is the minimum measurement of the key performance indicator (KPI) of airport capacity during a disruption. $RAPI_{KPI}^{DP}$ refers to the average slope of the KPI’s decline changes in the disruptive state, $RAPI_{KPI}^{RP}$ can be approximated by the average slope of the KPI recovering to the new steady state, *TAPL*_*KPI*_ is the time average loss of the KPI and *RA*_*KPI*_ is the recovering capacity of the KPIs.

## 3. Results

The data used in this study were obtained from VarFlight website, which included data from 232 airports in the mainland China and 82 airports in Europe. In this study, we selected the airports in China and Europe to perform a comparison since Europe is comparable in size to China. In addition, we obtained the flights data of Chinese and European airports for 12 months, from January 2020 to December 2020. The data pertaining to Chinese airports used in this study were obtained from the Civil Aviation Administration of China (CAAC), whereas those pertaining to European airports were obtained from the EUROCONTROL website. It is noteworthy the aircraft movements data contained cargo flight data. Additionally, 11 airports were selected from 30 million airports in China, and the top-8 airports in Europe were selected from the world’s top 50 airports in term of throughput in 2019. The top-8 airports in Europe represent major national hubs with a significant level of pan-regional and international traffic. Data of confirmed cases of COVID-19 in China and Europe were obtained from the Center for System Science and Engineering at Johns Hopkins University. We primarily obtained the data for mainland China, France, Germany, Netherlands, Spain, and the United Kingdom in 2020.

### 3.1 Vulnerability assessment of airport network

**Fig 3:**
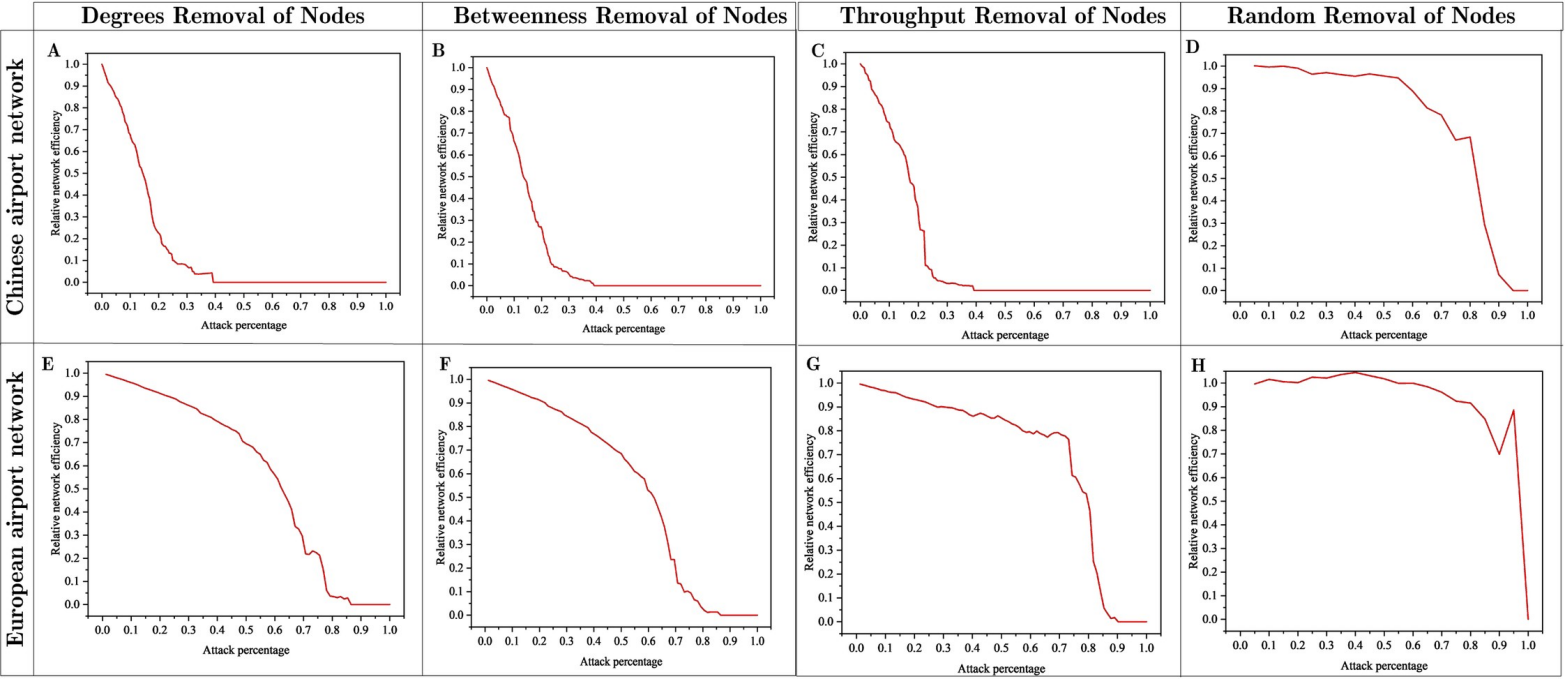
Efficiency comparison between Chinese and European airport networks based on different attack strategies.

Fig 3 shows the response of the CAN and EAN to node removal based on four attack strategies: degree removal, betweenness removal, throughput removal, and random removal. As shown from Fig 3, when the network efficiency began to decline, the decline speed of the random-removal strategy in the CAN was slower than those of three attack strategies. For centrality-based attacks (degree and betweenness) and strength (throughput) attacks, the CAN’s efficiency decreased abruptly initially and then declined to zero after 40% of the highly connected or central nodes were removed (see Fig 3(A)–3(C)). However, Fig 3(E)–3(G) show that after approximately 90% of the high degree, high betweenness, or high throughput nodes were removed, the EAN’s efficiency is reduced to zero. This implies that the connectivity of the EAN was greater than that of the CAN; as such, it is more robust and tends to have fewer failures when and disruption occurs. The authors of [34] proposed airport networks that are inherently resilient to random nodes or edge failures. In those networks, even when a significant number of nodes are removed, all integrity measures decreased only slightly and did not reach a sharp threshold, subsequently, the networks were completely destroyed. Fig 3(D) and 3(H) show that the CAN and EAN exhibited favorable invulnerability toward random removal attacks; however, it appeared that they were relatively vulnerable to selective attacks. However, we re-emphsize that this study focuses on the method of resilience assessment for airport networks subjected to global-scale hazards. It is noteworthly that although selective attacks can result in a higher collapse rate, two network metrics can be used to measure the degree and betweenness. In addition, we used selective attacks to trigger the collapse of global-scale disruptive events because pandemics, such as the COVID-19, affect the hub airports/facilities first. In general, the analysis results based on attack strategies showed that the CAN’s vulnerability was relatively low.

### 3.2 Comparison of different recovery strategies

**Fig 4:**
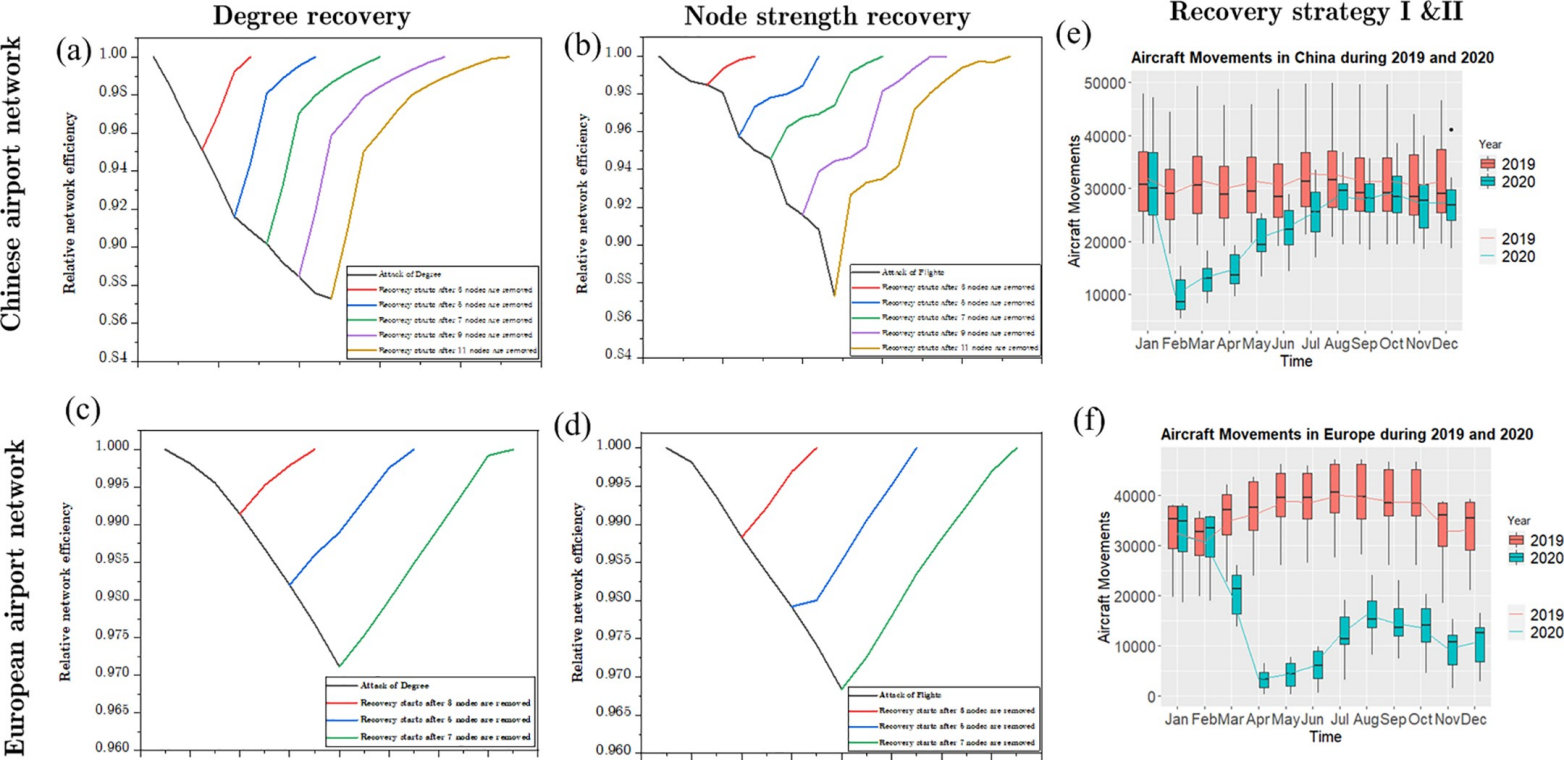
Comparison of network efficiency based on different recovery strategies. (a)–(d) show the recovery process of the CAN and EAN based on degree and node strength recovery strategies; the red box and blue box in (e) and (f) represent variation in air traffic of airports in China and Europe in 2019 and 2020, respectively.

From a complex network perspective, we simulated the attack-recovery process to analyze the effects of the CAN and EAN node recovery on the network efficiency. We selected degree and node strength as the recovery strategies for the recoveries after attack 1, 3, 5, 7, and 9 nodes, respectively. Fig 4(A)–4(D) show that the recovery time of failure nodes in the network directly affected the network efficiency. In addition, the path to optimal recovery is vital to the network resilience and should be investigated in future research.

From the view of actual operation data, Fig 4(E) and 4(F) present the actual recovery effects of the CAN and EAN during the COVID-19 pandemic. China and Europe have undergone the most significant changes in aircraft movements in 2020. We observed that the operation of domestic airports in China resumed to the pre-epidemic level because the Chinese authorities adopted strict epidemic prevention policies (recovery strategy I). However, the air traffic of European airports was less than half of that prior to the epidemic. This shows that the wider variation and associated fragmentation of national response actions in terms of travel restrictions and lock-downs imposed a more substantial effect on the operation of airport networks. China demonstrated a higher level of recovery as a single country under a single policy.

**Fig 5:**
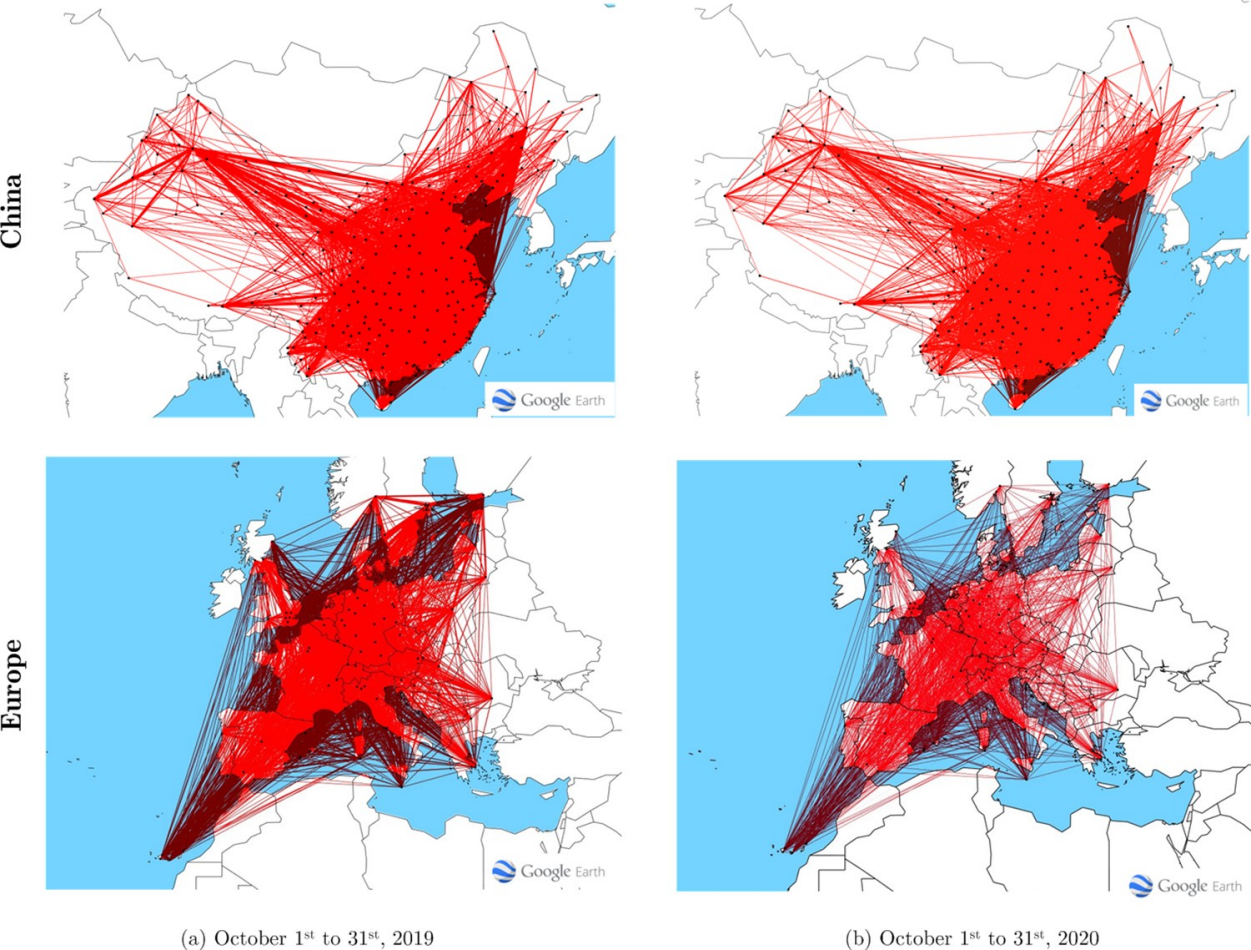
Comparison between CAN and EAN on October 1^st^ to 31^st^ before and during the COVID-19 pandemic.

Fig 5 shows the CAN and EAN on selected dates before and throughout the COVID-19 pandemic. These images show the connectivity patterns between airports, where the links represent the direct flights between them. As shown, the domestic air traffic in China in October 2020 did not changed significantly compared with the same period in 2019; the average degree centrality and average weighted degree of the CAN in 2020 increased by 2.265% and 5.9% compared with those in 2019, respectively. Most of the domestic airports in China have mostly recovered to their original connectivity, which may due to the strict epidemic prevention policy(Recovery strategy I). By contrast, the European air traffic in October 2020 changed significantly compared with the same period in 2019, and the connectively of the EAN was relatively sparse in October 2020. The average degree centrality and average weighted degree of the EAN in 2020 are reduced by 20.9% and 63.7% compared with those in 2019. Therefore, from a complex-network perspective, the variation in approaches across different European countries to contain the spread of the COVID-19 and the associated travel constraints affected the European network. The images presented above show that a specific reason contributing to the transportation network’s targeted attacks (restrictions on air passengers’ mobility) is difficult to identify. However, the proactive epidemic prevention and control policy (recovery strategy I) of a country/region can significantly affect its airport recovery. However, the less strict epidemic control policy (recovery strategy II) did not significantly affect the recovery rate of the airports.

### 3.3 Resilience assessment

In this section, we present results obtained from the node-level perspective. This paper selected a resilience metric to assess the operational resilience of individual airports during the COVID-19 epidemic.

**Fig 6:**
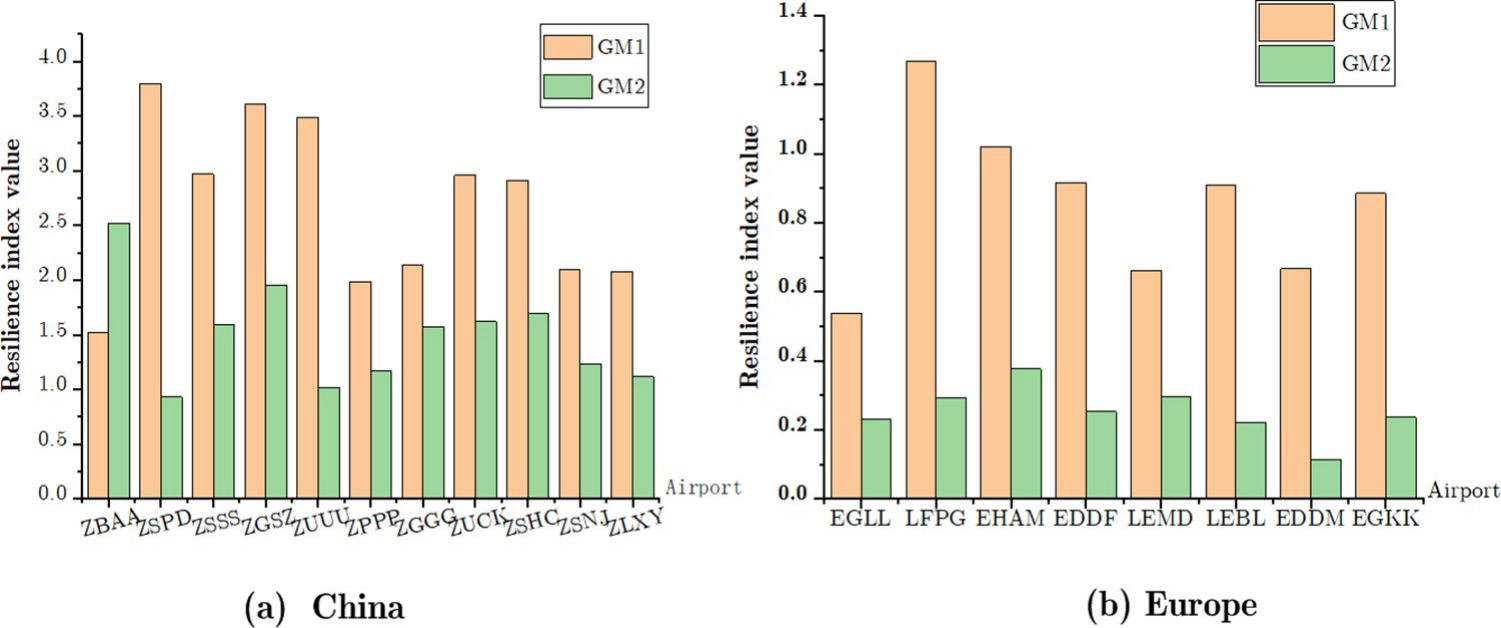
Resilience index of airports during COVID-19 pandemic. Orange and green bar represent the resilience metrics of the first seven months of 2020 and entire year of 2020, respectively.

Fig 6 shows the resilience value for selected airports from the CAN and EAN in the first seven months and the entire years of 2020 during the epidemic. In the first wave of the COVID-19, China has adopted active prevention policies (Recovery **strategy I)** to curb the spread of the epidemic. A comparison with other airports in the mainland China indicated that the resilience metric of the Shanghai Pudong International Airport(ZSPD) aircraft movements was the highest during the first seven months of 2020. Owing to cases from abroad in the second half of 2020, the average value of resilience metric in the selected Chinese airports decreased from 2.69 to 1.49 (see Table 2 and Fig 6A). However, for the European airports that adopted recovery strategy II and travel ban restrictions between countries, the resilience metric was lower during the first seven months of 2020 compared with that of China. For instance, the Paris International Airport (LFPG) performed relatively well in terms of recovery among the selected European airports. In addition, the operation of European airports has been severely affected by the second wave of the epidemic in the second half of 2020. Hence, the resilience of aircraft movements in Europe decreased significantly. The average resilience value was only 0.25 (see Table 2 and Fig 6B). This shows that the recovery of network- and airport-level demands in China and Europe differed significantly. The air transport lock-down execution time in different countries/regions was not synchronized, different epidemic prevention strategies were used, and the strategic perspective on the recovery of air traffic worldwide varied.

**Table 2:** Comparison of resilience metrics in China and Europe during COVID-19 epidemic.

Recovery Strategy I			Recovery Strategy II		
Airport	GM1	GM2	Airport	GM1	GM2
Beijing (ZBAA)	1.5188	2.5212	Heathrow (EGLL)	0.5386	0.2314
Pudong (ZSPD)	3.7932	0.9294	Paris (LFPG)	1.2667	0.2944
Hongqiao (ZSSS)	2.9686	1.5914	Amsterdam (EHAM)	1.0211	0.3767
Shenzhen (ZGSZ)	3.6086	1.9516	Frankfurt (EDDF)	0.9164	0.2529
Chengdu (ZUUU)	3.4847	1.0221	Madrid (LEMD)	0.6618	0.2950
Kunming (ZPPP)	1.9877	1.1724	Barcelona (LEBL)	0.9103	0.2229
Guangzhou (ZGGG)	2.1367	1.5741	Munich (EDDM)	0.6674	0.1137
Chongqing (ZUCK)	2.9576	1.6206	Gatwick (EGKK)	0.8848	0.2374
Hangzhou (ZSHC)	2.9096	1.6997			
Nanjing (ZSNJ)	2.0976	1.2365			
Xian (ZLXY)	2.0776	1.1161			

Note: GM1 and GM2 represent aircraft movements’ resilience metrics in airports during the first seven-month and the whole year of 2020, respectively.

### 3.4 Resilience improvement path of airport network

The implementation of various traffic mode and travel policies has recovered domestic travel in many countries; however international traffic remains restricted. The wider variation and associated fragmentation of national response actions have significantly affected the recovery of operational efficiency of the airport network. In addition, the current airport networks are highly structured and regulated; hence, the resilience improvement of airport networks affected by an epidemic should be considered from a system perspective. First, industry–government collaborations should be strengthened, such as the introduction of global vaccination passports [35]. Next, a global governance system for the aviation industry should be established to manage a worldwide emergencies. Finally, national civil aviation departments should enhance worldwide cooperation and establish an emergency management synergy mechanism to manage global-scale disruptive events. Methods to improve the resilience of airport networks should be furtther analyzed and investigated in future studies.

## 4. Conclusion and discussion

In this study, we proposed and tested a framework to assess the resilience of the airport network. The assessment framework contained the indicators shown in Fig 2. First, we assessed the vulnerability of airport networks based on different attack strategies from a complex network-level perspective. Subsequently, different recovery strategies used for airport networks were evaluated. Next, from a node-level perspective, based on the resilience metric, the operational resilience of crucial nodes in airport networks was evaluated. Finally, we compared the vulnerability of the CAN and EAN, and then assessed the resilience of the crucial nodes. It was discovered that the connectivity and vulnerability of the CAN during selective attacks were lower than those of the EAN, indicating that the EAN was highly robust and continued to operate effectively. By contrast, the CAN’s efficiency decreased significantly to 50% after 10% of the nodes were removed. The design and development of the CAN should be analyzed at the network level. However, the CAN during the COVID-19 epidemic was sighnificantly more active as compared with the EAN, and its network efficiency was higher. Therefore, the CAN may benefit from a single country under a single policy. Moreover, from a local airport perspective, we use data to evaluate the resilience of crucial airports in China and Europe during the epidemic. The domestic airports in China recovered at a much higer rate than those in Europe. The results showed that although the network indicators were vital to the resilience assessment of an airport network affected by global disturbance and that the policy in actual operations should be considered.

In this study, a resilience assessment framework for the airport networks was construced. It provides new insights to decision-makers for strengthening infrastructure resilience, e.g., airport network response to global disruptive events and improved emergency response. Compared with the conventional network structure approach, the proposed evaluation approach is based on operational data, instead of assumptions, rendering the results more meaningful for real-world airport network operations. However, one should note that this study also has several limitations, which should be improved in future research. For example, our current analysis neither involve changes in the networks of other countries (e.g., the US airport network)before and after the pandemic nor the effects of node strength changes on a complex network. In this study, airport nodes’ aircraft movements were disregarded in the analysis, where connectivity measures were emphasized. More factors should be considered in future studies to analyze an airport network’s resilience improvement strategies for a large-scale emergencies.

## Supporting information

S1 File (RAR)
